# Heat Stress Effects on Milk Production and the Genomic Architecture of Thermotolerance in Dairy Cattle

**DOI:** 10.3390/biology15100813

**Published:** 2026-05-21

**Authors:** Qingshan Ma, Mohamed Tharwat, Fahad A. Alshanbari, Muhammad Zahoor Khan

**Affiliations:** 1 College of Agriculture and Biology, Liaocheng University, Liaocheng 252000, China; maqingshan@lcu.edu.cn; 2 Department of Clinical Sciences, College of Veterinary Medicine, Qassim University, P.O. Box 6622, Buraidah 51452, Saudi Arabia; atieh@qu.edu.sa; 3 Department of Medical Biosciences, College of Veterinary Medicine, Qassim University, P.O. Box 6622, Buraidah 51452, Saudi Arabia

**Keywords:** dairy cattle, heat stress, thermotolerance, mammary gland, milk biosynthesis, genomic selection

## Abstract

Heat stress (HS) is among the most economically consequential environmental challenges to global dairy production, causing progressive declines in milk yield, compositional quality, and mammary cellular integrity. The temperature–humidity index (THI) is the primary thermal load metric, with performance-impairment thresholds typically beginning at THI 68 in Holstein cattle, with severe impacts manifesting beyond THI 72; breed-specific thresholds for Jersey, Brown Swiss, and Simmental cows differ owing to their lower metabolic heat load and greater inherent thermotolerance. At the molecular level, HS activates heat shock protein networks—notably *HSPA1A*, *HSP90B1*, and *HSPH1*—through *HSF1*/*HSF4* transcriptional activation, while simultaneously suppressing casein genes (*CSN1S1*, *CSN2*, *CSN3*), lipogenic genes (*FASN*, *SCD*, *CD36*), amino acid transporters (*SLC7A5*, *SLC38A2*), and mTOR-AKT-STAT5 translational machinery, collectively impairing milk biosynthetic capacity. Pro-apoptotic signaling (*BAX*, *CASP3* upregulation; *BCL2* downregulation) and mitochondrial dysfunction further compromise mammary epithelial viability. Post-transcriptional regulation through miRNA, circRNA, and lncRNA competing endogenous RNA networks, alongside epitranscriptomic m6A modifications, adds further regulatory complexity. Genome-wide association studies have identified SNPs in *HSP70A1A*, *HSPA4*, *TLR4*, and *PRLR* as thermotolerance candidates compatible with sustained milk production. Nutritional supplementation with methionine, arginine, and taurine partially restores cellular synthetic capacity. Integrating multi-trait genomic selection with *Bos indicus* introgression, precision cooling, and targeted nutrition offers the most viable path toward climate-resilient, high-producing dairy cattle.

## Simple Summary

This narrative review integrates current evidence on the effects of heat stress on milk production and the genomic basis of thermotolerance in dairy cattle. Heat stress poses a critical and escalating threat to global dairy productivity, causing progressive declines in milk yield, composition, and mammary cellular integrity as temperature–humidity index values exceed established thresholds. At the molecular level, thermal challenge activates heat shock protein networks while suppressing casein gene expression, lipogenic pathways, and mTOR-AKT-STAT5 signalling—collectively impairing milk biosynthetic capacity. Pro-apoptotic activation and non-coding RNA-mediated post-transcriptional dysregulation further compromise mammary epithelial viability, with consequences extending into subsequent lactations when thermal stress occurs during the dry period. Genome-wide association studies have identified SNP markers within heat shock protein genes, immune regulators, and thermoregulatory signalling pathway components conferring thermotolerance without adverse effects on milk production traits, while strategic *Bos indicus* genomic introgression offers a complementary breeding avenue. Targeted nutritional interventions partially restore cellular synthetic capacity and represent practical near-term mitigation tools under heat stress conditions. Integrating genomic selection, targeted nutrition, and precision cooling represents the most viable strategy for developing climate-resilient, high-producing dairy cattle.

## 1. Introduction

Global dairy cattle production is a cornerstone of agricultural economies worldwide, contributing over 900 million tonnes of milk annually and supporting the livelihoods of more than one billion people [1]. The genetic selection of high-yielding breeds, particularly Holstein-Friesian, has markedly increased milk output per animal; however, this productivity advantage is accompanied by elevated metabolic demands and a heightened vulnerability to environmental perturbations. Among these, heat stress (HS) stands out as one of the most economically and physiologically consequential challenges confronting the modern dairy industry [2].

HS arises when the cumulative thermal load from ambient temperature, relative humidity, solar radiation, and wind speed exceeds an animal’s capacity for heat dissipation, thereby disrupting homeothermy [3,4]. Dairy cattle maintain core body temperature within a narrow physiological range of 38–39.2 °C, and any sustained departure from this range triggers a cascade of adaptive and pathological responses that collectively impair productive performance [5]. The temperature–humidity index (THI) remains the most widely used metric for quantifying the combined thermal challenge posed by ambient temperature and relative humidity [6,7], with a consensus HS threshold of 68–72 THI units for Holstein cows; beyond this, measurable declines in dry matter intake (DMI), milk yield, and milk composition are consistently observed [8,9,10,11,12,13]. Notably, high-producing cows may exhibit performance losses at even lower THI values due to their elevated endogenous metabolic heat load from milk synthesis [14].

Although the THI 68–72 threshold has been most extensively characterized in Holstein-Friesian cattle, accumulating evidence demonstrates that breed-specific thresholds and response magnitudes differ markedly across dairy populations. Jersey cows generally exhibit greater inherent thermotolerance than Holsteins, attributed to their smaller body size, lower absolute metabolic heat production, and more efficient evaporative heat dissipation; comparative studies in Korea reported that Jersey cows maintained more stable physiological parameters and milk production than Holsteins under equivalent thermal challenge [15]. Hungarian Simmental and other dual-purpose breeds similarly demonstrate attenuated declines in milk yield and composition compared with Holstein–Friesians under summer heat exposure, with Holsteins showing the largest absolute losses among the three breeds tested [16]. Persistent heat-stress effects have also been documented in Simmental cows in continental European climates (Eastern Croatia), where elevated THI during early lactation produced measurable lag effects on subsequent test-day yields [17]. Brown Swiss cattle have likewise been reported to display divergent milk microbiota and compositional responses relative to Holsteins under identical thermal conditions [18], collectively underscoring that breed selection itself constitutes a meaningful axis of thermotolerance and that universal application of a single Holstein-derived THI threshold across global dairy populations would substantially misrepresent breed-specific risk.

**Figure 1:**
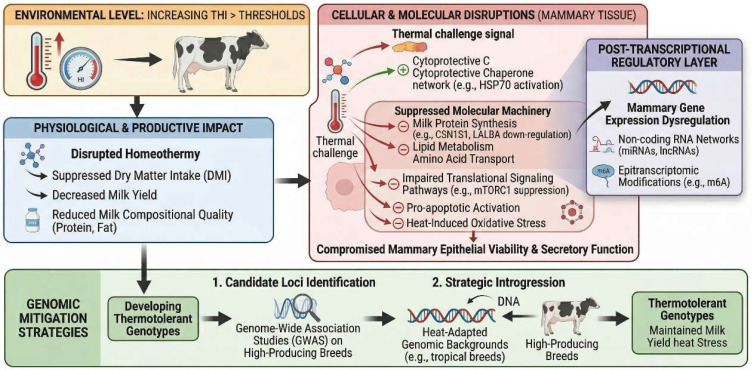
Integrated overview of heat stress-induced physiological and molecular disruptions and genomic mitigation strategies in dairy cattle.

At the molecular level, HS activates the highly conserved heat shock response, driven principally by transcription factors *HSF1* and *HSF4*, upregulating molecular chaperones—including *HSPA1A*, *HSPH1*, *HSP70*, *HSP90B1*, and *DNAJB1*—to safeguard cellular proteostasis [19,20,21]. This cytoprotective response, however, incurs a substantial cost to mammary synthetic capacity: milk protein synthesis is impaired through converging transcriptional and post-transcriptional mechanisms—including downregulation of casein genes (*CSN1S1*, *CSN2*, *CSN3*, and *CSN1S2*) and amino acid transporters (*SLC7A5*, *SLC38A2*, *SLC38A3*), alongside reduced phosphorylation of *STAT5* and *S6K1* and attenuation of *mTOR*/*AKT* signaling—collectively constraining both substrate supply and translational initiation capacity [22,23]. Concurrently, de novo lipogenic genes—including fatty acid synthase (*FASN*), stearoyl-CoA desaturase (*SCD*), and the long-chain fatty acid uptake transporters *CD36* and *SLC27A6*—are significantly attenuated, contributing to the characteristic decline in milk fat yield consistently observed under thermal stress conditions [19,24]. These molecular disruptions are further compounded by heat-induced oxidative stress and pro-apoptotic activation, encompassing upregulation of *BAX*, *CASP3*, and *CASP9* and suppression of *BCL2*, which collectively compromise mammary epithelial viability and secretory function [25]. The pronounced inter-individual and inter-breed variation in the magnitude of these molecular disruptions—and in overall thermotolerance—has provided the scientific impetus for genetic selection targeting heat tolerance while concurrently maintaining milk production performance in cattle. Genome-wide association studies (GWAS), transcriptomic profiling, and quantitative trait loci (QTL) mapping have collectively identified genetic markers—encompassing single-nucleotide polymorphisms (SNPs) and differentially expressed genes—that discriminate thermotolerant from thermosensitive genotypes and serve as promising targets for genomics-assisted breeding [26,27]. Candidate loci of particular significance include SNPs within *HSP70A1A*—encompassing a promoter variant (g.-133 G>C) that enhances chaperone expression and a coding variant (g.462 G>T) altering protein structure—alongside *HSPA4* (g.44660065 C>T in the 3′ UTR), the latter associated with improved thermotolerance without compromise to milk production traits, the prolactin receptor (*PRLR*), and immune and signaling pathway genes *TLR4* and *SMAD3*—collectively associated with enhanced thermoregulatory capacity without disproportionate sacrifice of milk yield [26,28,29]. Strategic introgression of *Bos indicus* genomic segments into *Bos taurus* genetic backgrounds represents an additional genomic avenue for simultaneously improving heat resilience and preserving productive potential [30]. Emerging evidence further implicates non-coding RNA networks—comprising microRNAs (including bta-miR-21-5p, bta-miR-146b, and miR-223), circular RNAs (*circ_002033*, *circFCHSD2*), and long non-coding RNAs (*MSTRG.8646.1*, *MSTRG.6147.5*) functioning as competing endogenous RNA (ceRNA) sponges—alongside epitranscriptomic N6-methyladenosine (m6A) modifications of key HS-responsive genes including *HSP70*, *HSF1*, *BAX*, and *CASP3*, as post-transcriptional regulatory determinants of mammary epithelial dysfunction and impaired milk synthesis that further shape the genomic architecture of heat tolerance [31,32,33,34]. Collectively, these findings underscore the scientific and practical feasibility of developing integrated selection indices that simultaneously advance heat resilience and sustain milk production in increasingly thermally challenging production environments (Figure 1).

Despite substantial progress across these physiological, molecular, and genomic dimensions, an integrated perspective that bridges whole-animal productivity metrics, gene-expression networks, and genomic marker discovery remains lacking in the literature. Such integration is needed to inform multi-pronged mitigation strategies encompassing precision nutrition—including bioactive compounds and antioxidant supplementation that can attenuate heat stress-induced oxidative distress through Nrf2 signaling activation [35]—strategic cooling management, and genomics-assisted selection for climate-resilient genotypes. The present narrative review therefore aims to: (i) discuss the effects of HS on milk production performance and composition in dairy cows; (ii) examine the molecular mechanisms by which HS disrupts milk synthesis-related gene expression; and (iii) provide an overview of genetic and genomic markers associated with HS resistance, with particular emphasis on their application in marker-assisted and genomic selection programs. The distinctive contribution of the present review, relative to existing heat-stress reviews, is fourfold: (a) it integrates THI thresholds, mammary gene-expression networks, ncRNA/m6A regulatory layers, and SNP-level genomic markers within a single THI → cellular stress → gene regulation → phenotype scaffold rather than treating these layers as parallel literature; (b) it explicitly reconciles methodological inconsistencies in the field, including the apparent mTOR up- versus down-regulation paradox and the discrepancy between MAC-T-derived and in vivo transcriptomic responses; (c) it foregrounds breed-specific differences (Holstein, Jersey, Brown Swiss, Simmental, Bos indicus) and their implications for the generalizability of Holstein-derived molecular and SNP evidence; and (d) it links each major molecular cluster to actionable mitigation entry points (precision cooling, targeted amino acid supplementation, multi-trait genomic selection), so that the review functions as a bridge between mechanistic biology and on-farm decision-making rather than as a bibliographic catalogue. By narratively integrating evidence across physiological, transcriptomic, and genomic scales, this review seeks to advance the conceptual framework for breeding high-producing, climate-resilient dairy cows in an increasingly thermally challenging global environment. To make this integration explicit, we adopt throughout the review a unifying THI → cellular stress → gene regulation → phenotypic outcome framework, in which rising THI imposes a thermal load that exceeds the cow’s evaporative and behavioral dissipation capacity, triggering coordinated cellular stress responses (HSF1/HSF4-driven HSP induction, ER stress, mitochondrial dysfunction, oxidative imbalance, and pro-apoptotic signaling). These cellular events propagate into transcriptional and post-transcriptional reprogramming—suppression of casein and lipogenic gene networks, attenuation of mTOR-AKT-STAT5 translational machinery, and ncRNA/m6A-mediated fine-tuning of HS-responsive transcripts—which collectively manifest as the phenotypic endpoints of reduced milk yield, altered milk composition, impaired mammary involution and redevelopment, carry-over losses into the next lactation, and developmental programming of offspring performance. Genomic markers in HSP, immune, and signaling genes intersect this framework as inter-individual modifiers explaining why genetically similar cows differ in thermosensitivity, and therefore form the natural targets for marker-assisted and genomic selection. This conceptual scaffold is referenced explicitly in each subsequent section.

Figure 1 presents the schematic overview of the impact of heat stress (HS) on dairy cattle productivity and the corresponding molecular, genomic, and nutritional mitigation landscape. At the environmental level, increasing temperature–humidity index (THI) values beyond established thresholds disrupt homeothermy in high-producing dairy cows, triggering adaptive and pathological responses that progressively suppress dry matter intake, milk yield, and milk compositional quality. At the cellular level, thermal challenge activates cytoprotective chaperone networks while suppressing the molecular machinery governing milk protein synthesis, lipid metabolism, and amino acid transport, with attenuation of translational signalling pathways, pro-apoptotic activation, and oxidative stress that compromise mammary epithelial viability and secretory function. A post-transcriptional regulatory layer comprising non-coding RNA networks and epitranscriptomic modifications further modulates mammary gene expression under thermal challenge. At the genomic level, candidate loci identified through genome-wide association studies, alongside strategic introgression of heat-adapted genomic backgrounds into high-producing breeds, provide tractable targets for developing thermotolerant genotypes; however, their effects must be interpreted in light of the negative genetic correlation between thermotolerance and yield and the genotype-by-environment interactions documented across temperate and tropical populations.

## 2. Literature Search Strategy

This narrative review draws on peer-reviewed literature retrieved from PubMed, Web of Science, Scopus, and Google Scholar, with supplementary records from FAO and ScienceDirect. The primary search window was January 2020 to May 2026; a limited number of seminal earlier publications (dating back to 2014) were retained where necessary to provide foundational context and to streamline discussion of mechanistically relevant content. Search terms combined “heat stress,” “thermotolerance,” “dairy cattle,” “milk yield,” “milk composition,” “mammary gland,” “heat shock protein,” “HSF1,” “casein,” “mTOR,” “non-coding RNA,” “m6A,” “GWAS,” “SNP,” and “*Bos indicus*/*Bos taurus*” via Boolean operators (AND/OR). Reference lists of retrieved articles were hand-screened for additional sources. Priority was given to original research, transcriptomic and genomic studies, meta-analyses, and authoritative reviews published in English in Science Citation Index (SCI)-indexed journals; conference abstracts, non-peer-reviewed reports, and studies lacking methodological transparency were excluded. Consistent with the narrative scope of this review, no formal quality scoring, risk-of-bias assessment, or PRISMA-compliant screening was undertaken; sources were selected based on topical relevance, scientific rigor, and contribution to the integrative THI → cellular stress → gene regulation → phenotype framework adopted herein.

## 3. Effect of Heat Stress on Milk Performance and Milk-Related Associated Markers in Dairy Cattle

Heat stress (HS) represents one of the most economically significant challenges to modern dairy production, impairing milk yield, compositional quality, and metabolic efficiency through a cascade of interrelated physiological and endocrine disruptions [36,37]. The following sections examine the dose–response dynamics of HS on milk output and quality, the biological and genetic factors that modulate individual cow susceptibility, and the temporal, herd-level, and environmental consequences of sustained thermal challenge. The effect of heat stress on milk performance has been summarized in Table 1. The studies summarized in Table 1 vary considerably in THI thresholds, exposure duration, lactation phase, parity, and breed composition; values are therefore presented as a qualitative inventory of reported effects rather than as directly comparable quantitative estimates.

### 3.1. Quantitative Impact of Heat Stress on Milk Yield and Quality: Dose–Response and Threshold Effects

Understanding the magnitude of HS-induced milk losses requires moving beyond isolated observations toward a structured, dose–response framework that accounts for threshold effects, biological modifiers, and temporal dynamics. The temperature–humidity index (THI) has emerged as the standard metric for quantifying the thermal load experienced by dairy cattle, and substantial evidence now confirms that its relationship with milk production is both linear and progressive beyond critical thresholds. Optimal milk yield is observed within a THI range of 60–73; when THI exceeds 73, milk production declines substantially, with losses of approximately 5–8% relative to production at thermoneutral conditions [38]. This threshold is not universal across production environments or genetic backgrounds, but its existence is remarkably consistent across diverse geographic and management systems.

The definition of the HS threshold varies modestly by region, breed composition, and production system, yet the directionality of the response remains invariant. In Canadian Holstein cow populations across over 387,000 test-day records, the average threshold for milk-yield decline was identified at THI~max~ 68 (or THI~avg~ 64), with fat and protein components responding even earlier—at THI~max~ 57 and THI~max~ 60, respectively—demonstrating that milk quality is more thermally sensitive than milk quantity [39]. This dissociation between quality and quantity thresholds is of considerable practical significance, as economic losses may accrue through compositional deterioration before any volumetric decline becomes apparent. Consistent with this, on Holstein-dominated farms in northern Italy, milk yield remained statistically unaffected by either THI or maximum daily THI. In contrast, fat, protein, and somatic cell scores were significantly reduced, confirming that quality deterioration at lower THI values drives economic losses in temperate dairy regions [40].

A meta-analysis of 16 studies in which dairy cattle were exposed to THI > 72 demonstrated a mean effect size of 50% reduction in milk yield, with a significant linear relationship between increasing THI and declining production (*p* < 0.0001) [41]. When translated to the farm level, the dose–response relationship becomes particularly evident under tropical and subtropical conditions. In Holstein–Friesian cattle in Mexico, milk yield declined progressively from 26.5 kg/day under non-HS conditions (THI < 68) to 15.0 kg/day under intense HS (THI ≥ 77)—a reduction of approximately 43%—accompanied by declines in milk fat from 3.9% to 3.0% and protein from 3.3% to 2.6%. At the same time, somatic cell count doubled from 200,000 to 400,000 cells/mL [42]. In Thai–Holstein cattle, the HS threshold was identified at THI 76, above which milk yield declined at a rate associated with the proportion of Holstein genetics, suggesting that high-merit crossbred animals carrying greater Holstein ancestry may paradoxically exhibit greater thermosensitivity [43]. Similarly, in Sahiwal cows, the highest milk yield was recorded during winter (THI: 60), with the lowest during hot humid summer (THI: 81), and a strong negative correlation was observed between THI > 79 and milk yield, fat, and lactose [44].

A fundamental quantitative benchmark is provided by controlled experiments demonstrating that transferring a lactating Holstein cow from an ambient temperature of 18 °C to 30 °C reduces milk yield, milk fat, solids-not-fat, and protein percentages by approximately 15%, 39.7%, 18.9%, and 16.9%, respectively [45]. These figures underscore the disproportionate sensitivity of fat synthesis to thermal perturbation relative to other components. Under cyclical HS conditions (THI 76–80), voluntary dry matter intake (DMI) decreased by 35%, and this reduction in DMI accounted for 66% of the observed decline in milk yield; the remaining 34% was attributable to the direct metabolic effects of heat stress, independent of feed intake [46]. This partition of direct versus intake-mediated effects is critical: it establishes that even if nutritional strategies fully compensate for DMI depression, a substantial fraction of production loss would persist. Correspondingly, one of the direct effects of HS is reduced feed intake, which can lead to milk losses of 0.2–0.9 kg/day per cow [11].

Beyond volumetric loss, HS exerts compositionally selective effects on milk quality through distinct metabolic pathways. The reduction in milk protein and casein content under HS is primarily attributed to decreased protein synthesis in the mammary gland, as amino acids are redirected toward gluconeogenesis and other metabolic priorities to meet elevated energetic demands [47,48]. This decline in protein concentration impairs milk coagulation properties, compromising its technological suitability for processing [18]. Variations in milk fat content under HS are reported inconsistently in the literature—some studies describe an increase in fat percentage while others report a decrease—likely reflecting reduced availability of ruminal fermentation precursors such as acetate and butyrate resulting from altered ruminal microbiome activity [49,50]. Heat-stressed cows also exhibit significantly lower rumen pH, reduced rumen motility, decreased rumination time, and elevated rumen temperature compared with cooled contemporaries, as well as lower DMI (19.0 vs. 24.3 kg/d) [51]. Compared to pair-fed thermoneutral cows, heat-stressed animals showed a more pronounced decline in milk, fat, protein, and lactose yields over time, with absolute reductions of 3.9 kg/d for milk and 180, 186, and 205 g/d for fat, protein, and lactose yields, respectively, from day 3 to 14 of the heat challenge [52].

It is also important to acknowledge that, despite its widespread adoption, THI captures only ambient temperature and relative humidity and therefore omits several thermally relevant variables. Solar radiation, wind speed, infrared radiation from heated surfaces, and animal-level factors such as coat color, parity, body condition, and current production level are not represented in the standard THI equation. Yet, each materially affects the actual heat load experienced by an individual cow. As a result, two herds at the same THI but exposed to different solar loads or air movement can experience very different physiological strain. More refined indices have been proposed to address these gaps—including the Black Globe-Humidity Index (BGHI), the Heat Load Index (HLI) developed for feedlot cattle, the Comprehensive Climate Index (CCI), and the Equivalent Temperature Index for Cattle (ETIC)—alongside individual-cow biomarkers such as rectal temperature, respiration rate, panting score, and HSP70 expression. Continuous monitoring through wearable sensors (rumen boluses, ear-tag temperature loggers) and behavioral metrics (rumination time, panting) further complements THI by capturing the cow’s integrated thermal experience in real time. THI therefore remains a valuable population-level metric, particularly for retrospective and large-scale analyses, but at the individual-cow and farm-management level, it should be regarded as a screening tool rather than a definitive descriptor of heat stress. A formal meta-analysis or forest plot synthesizing milk-yield decline per THI unit across studies was not attempted here, given the substantial heterogeneity in THI definitions (e.g., THIavg vs. THImax), exposure duration, lactation stage, parity, breed composition, and reporting of variance estimate across the available literature. Such a quantitative synthesis remains an important priority for future research once primary studies adopt more standardized protocols.

### 3.2. Biological and Genetic Modifiers of Heat Stress Susceptibility

The impact of HS on milk yield is not uniform across all cows; it is substantially modulated by lactation stage, parity, and genomic background. Cows in mid or late lactation demonstrate greater tolerance of mild HS compared to early-lactation cows; however, this tolerance advantage reverses under moderate or severe HS, at which point all lactation stages are similarly compromised [53]. This non-linear interaction between THI and lactation stage necessitates dynamic management strategies calibrated to both ambient thermal load and the lactation stage of individual animals. Furthermore, both primiparous and multiparous Holstein cows are adversely affected by seasonal heat stress, with cows calving in hotter months (THI: 70.30 in July versus THI: 59.45 in October) exhibiting significantly lower average daily milk yields; the reduction is associated with heat stress-induced alterations in serum oxidative and antioxidative indices, indicating that compromised oxidative status mediates at least part of the lactation performance decline [54]. In pasture-based automatic milking systems studied by Morales-Piñeyrúa et al., for each one-unit increase in THI, milk yield decreased by approximately 0.18 kg in primiparous and 0.40 kg in multiparous cows, with this effect lagging by three days—a finding the authors attributed to the cumulative nature of preceding thermal conditions on lactation performance [55].

Genomic architecture further modulates thermal sensitivity. In a single study of Thai–Holstein cattle, the heritability of milk yield under heat stress (THI 76) was estimated at 0.347 ± 0.032, with a genetic correlation of −0.24 between milk yield and fat-to-protein ratio, and decline rates positively associated with greater Holstein genetics [43]. However, heritability of heat tolerance is population-specific and context-dependent, varying with breed composition, THI threshold, statistical model, and environmental gradient; reported estimates in other populations range more broadly (~0.05–0.34), reflecting differences in genetic background and genotype-by-environment interactions. This value should therefore be regarded as illustrative within its specific population and production context rather than as a generalizable parameter. Nonetheless, the consistent detection of non-zero heritabilities across populations confirms the tractability of genomic selection for thermotolerance. A distinction between heat-tolerant and heat-sensitive phenotypes has been experimentally validated, with heat-tolerant cows exhibiting significantly lower milk-yield losses (*p* = 0.0007) and lower Pearson correlation coefficients between THI and milk yield than heat-sensitive counterparts [56]. These findings provide the empirical foundation for genomic-assisted identification of thermotolerant individuals and population-specific selective breeding strategies.

### 3.3. Temporal Vulnerability Windows, Herd-Level Losses, and Environmental Consequences

Among the most consequential manifestations of HS is its capacity to exert persistent carry-over effects on milk production well beyond the acute exposure period, particularly when thermal stress occurs during the dry period or early lactation. Heat stress during the close-up dry period is associated with decreased lactation performance as a function of increasing THI at 21, 14, and 7 days before calving, resulting in milk yields of approximately 2.30, 2.60, and 2.90 kg, respectively, in the subsequent lactation [57]. Similarly, increasing THI from the day of calving through days +7, +14, and +21 post-calving was associated with decreased milk yield by 3.20, 4.10, 5.60, and 5.60 kg, respectively, in Polish Holstein–Friesian cows, with the greatest reduction coinciding with heat stress at +14 to +21 days post-calving—a critical window during which the metabolic and hormonal programming of peak lactation occurs [58]. These heat-stress effects during early lactation were accompanied by higher levels of leptin, nonesterified fatty acids, and β-hydroxybutyrate, indicating heat-stress-induced lipolysis and negative energy balance. Cows that experienced no heat stress (THI < 70) during the dry period produced significantly higher 305-day milk (10,926 ± 1206 kg) compared to those under moderate (THI 70–80: 10,799 ± 1254 kg) or severe (THI > 80: 10,691 ± 1297 kg) heat stress [59].

The temporal dimension of HS vulnerability extends further to prenatal programming. Maternal exposure to HS during pregnancy negatively affects the future milk production of offspring, with the timing of in utero exposure being the critical determinant: offspring exposed to heat stress only during the first trimester (HSE1) had the lowest milk production, while those exposed only during the third trimester (HSE4) had the highest production, establishing the early gestational period as the most critical window for developmental programming of lifetime lactation potential [60].

At the herd level, the aggregate production losses from HS substantially exceed estimates based on any single pathway. Direct reduction in milk yield decreased herd-level output by up to 6.9% over four months; however, when aggregated with heat-induced declines in reproductive efficiency and health, total milk-yield loss reached 8.6%, indicating that single-pathway analyses consistently underestimate the true production cost of HS [2]. A significant increase in both milk production and dry matter intake with alleviation of heat stress has been consistently demonstrated in Holstein lactating dairy cows [61,62], reinforcing the productivity dividend achievable through targeted cooling interventions. In Sudano-Guinean climate conditions, cows with cortisol levels above 11.7 ng/mL produced substantially less milk (7.81 L/day) compared to unstressed contemporaries (19.9 L/day), with production declining progressively as THI increased, linking endocrine stress biomarkers directly to production outcomes [63].

HS also degrades the environmental efficiency of dairy production. Heat-stressed cows exhibited lower dry matter intake and reduced milk yield relative to cooled contemporaries, and while absolute enteric methane (CH_4_) emissions were slightly lower under HS (326.4 vs. 351.8 g/d), CH_4_ intensity—expressed per kilogram of energy-corrected milk (ECM)—increased significantly (11.3 vs. 9.6 g/kg ECM), demonstrating that HS worsens the environmental footprint per unit of useful product [51]. This finding was corroborated in an independent study where heat stress increased enteric methane intensity by up to 6.6%, with reduced feed efficiency (up to 19.2%), further exacerbating methane intensity by an additional 4.4% [2]. Milk yield and feed efficiency progressively declined with the duration of heat stress exposure up to 20 days, and reductions in absolute methane emissions, methane yield, or methane intensity occurred at the expense of decreased milk yield and feed efficiency—outcomes incompatible with sustainable dairy production goals [64]. These findings collectively establish that mitigating HS is not merely a productivity imperative but also an environmental one, particularly under trajectories of global warming that will extend both the severity and duration of thermal challenges to dairy systems worldwide.

**Table 1:** Qualitative overview of reported effects of heat stress on milk yield, composition, feed intake, and related indicators in dairy cattle.

Breed	Country	THI/HS Conditions	Biological Effects (Milk Yield, Milk Composition, Feed Intake & Others)	Reference
Holstein	Canada	THImax threshold: 68; Fat threshold: THImax 57; Protein threshold: THImax 60	Progressive milk-yield decline per THI unit above threshold; Milk fat and protein more thermally sensitive than yield; Quality deterioration precedes volumetric decline; Feed intake indirectly reduced	[39]
Holstein	Northern Italy	THI and maximum daily THI assessed across seasons	Milk yield: statistically unaffected by THI; Milk fat and protein significantly reduced; Somatic cell scores elevated; Quality losses precede yield losses; Feed intake mildly reduced at moderate THI	[40]
Holstein (meta-analysis)	Multiple countries	THI > 72	Mean milk-yield reduction: 50%; Respiratory rate elevated (+65%); Rectal temperature elevated (+38%); Feed intake consistently reduced across studies	[41]
Holstein–Friesian	Mexico	THI < 68 (non-HS) to THI ≥ 77 (intense HS)	Milk yield: 26.5 → 15.0 kg/day (↓43%); Milk fat: 3.9 → 3.0%; Milk protein: 3.3 → 2.6%; SCC doubled: 200,000 → 400,000 cells/mL; Feed intake reduced	[42]
Thai–Holstein (crossbred)	Thailand	HS threshold at THI 76	Milk-yield heritability: 0.347 ± 0.032; fat-to-protein ratio altered; higher Holstein genetics → greater susceptibility to HS-induced milk loss; Feed intake reduced	[43]
Sahiwal (*Bos indicus*)	India (tropical semi-intensive)	Winter THI: 60 vs. Hot humid summer THI: 81	Highest milk yield in winter; lowest yield in hot humid season; milk fat and lactose negatively correlated with thi > 79; strong negative thi-milk yield correlation; feed intake seasonally reduced	[44]
Holstein	US	THI 76–80 (cyclical HS)	DMI ↓35%; 66% of milk-yield decline attributed to reduced feed intake; milk protein, fat, and solids yield lower; 34% of milk-yield decline independent of feed intake; rumen pH reduced; rumination time decreased	[46]
Holstein	Canada	34 °C ambient; THI ~ 83; up to 20 days exposure	Milk yield: ↓3.9 kg/d (days 3–14); milk fat: ↓180 g/d; milk protein: ↓186 g/d; milk lactose: ↓205 g/d; greater decline vs. pair-fed thermoneutral controls; feed intake reduced	[52]
Holstein	Uruguay	THI ≥ 68	Milk yield: ↓0.18 kg/THI unit in primiparous; ↓0.40 kg/THI unit in multiparous; 3-day lag effect of cumulative thermal load; feed intake reduced; effect lagged by preceding thermal conditions	[55]
Polish Holstein–Friesian	Poland	THI at calving +7 to +21 days post-calving	Milk yield: ↓3.20–5.60 kg/d; greatest reduction at +14–21 d post-calving; elevated NEFA, BHB, and leptin indicating lipolysis and negative energy balance; feed intake reduced	[58]
Holstein	Mexico	THI < 70, 70–80, >80 during dry period	305 d milk yield: 10,926 ± 1206 kg (no hs) vs. 10,799 ± 1254 kg (moderate hs) vs. 10,691 ± 1297 kg (severe hs); feed intake during dry period reduced; carry-over effects on subsequent lactation	[59]
Holstein vs. Jersey (comparative)	South Korea	Summer THI elevation in lactating cows	Holsteins exhibited greater physiological perturbation (rectal temperature, respiratory rate) and larger declines in milk yield than Jerseys under identical conditions; Jersey cows displayed greater inherent thermotolerance attributed to lower body mass and more efficient evaporative dissipation	[15]
Holstein–Friesian, Jersey, Hungarian Simmental	Hungary	Summer heat exposure across breeds	Holstein–Friesians displayed the largest reductions in milk yield and milk solids; Jersey and Hungarian Simmental cows showed attenuated responses, demonstrating breed-specific susceptibility and supporting inclusion of breed in any HS risk model	[16]
Simmental	Eastern Croatia	Elevated THI during early lactation (continental climate)	Persistent (lag) effects of early-lactation HS on subsequent test-day milk yield in Simmental cows; effect persists weeks after acute thermal exposure ends, paralleling lag effects reported in Holsteins	[17]
Sudano-Guinean cattle	Cameroon (suburban area)	Cortisol > 11.7 ng/mL as HS marker; rising THI conditions	Milk yield: 7.81 L/d (stressed) vs. 19.9 L/d (unstressed); progressive decline with rising THI; cortisol validated as HS biomarker; feed intake reduced under high-cortisol conditions	[63]

THI = Temperature–Humidity Index; HS = Heat Stress; DMI = Dry Matter Intake; SCC = Somatic Cell Count; NEFA = Non-Esterified Fatty Acids; BHB = Beta-Hydroxybutyrate; “↓” indicates decrease.

Take-home patterns from Table 1: across heterogeneous geographies, breeds, and study designs, four directionally consistent qualitative trends emerge—noting that the absolute magnitudes reported vary substantially across studies and are not directly comparable in the absence of formal meta-regression. (i) Quality precedes quantity: milk fat and protein concentrations tend to decline at lower THI thresholds than milk yield itself, so economic losses through compositional deterioration generally begin before volumetric losses become apparent. (ii) Breed-dependent thermosensitivity: Holstein–Friesian and high-Holstein crossbreds appear consistently more thermosensitive than *Bos indicus* and indigenous tropical breeds (e.g., Sahiwal), the latter showing more graded, season-dependent declines. (iii) Dual mediation of yield loss: a substantial proportion of milk-yield loss is mediated by reduced DMI, while a non-trivial residual fraction reflects direct metabolic effects independent of feed intake, indicating that nutritional management alone cannot fully restore productivity. (iv) Carry-over effects: heat stress experienced during the dry period and early lactation extends measurably into subsequent lactation performance, so single-time-point estimates tend to underestimate the cumulative productivity cost of HS. These patterns should therefore be interpreted as the direction of effect rather than as quantitatively comparable estimates across studies.

The studies in Table 1 differ substantially in THI thresholds, exposure duration, lactation phase, parity, breed composition, and statistical methodology. Consequently, the reported effects cannot be directly compared across rows, and the table should be interpreted as a qualitative synthesis of the existing literature rather than a quantitative summary. A formal meta-regression integrating these moderators was beyond the scope of the present narrative review and represents an important direction for future work.

## 4. Genetic Markers Regulated in the Bovine Mammary Gland in Response to Heat Stress

Heat stress (HS) exerts a multifaceted molecular assault on the bovine mammary gland, simultaneously compromising cellular integrity, suppressing lactogenic gene networks, and redirecting transcriptional and translational resources toward cytoprotective pathways [25,31,32,33,34]. The molecular consequences of HS extend across multiple regulatory layers—from classical heat shock protein (HSP) induction and apoptotic signaling to epigenetic remodeling via non-coding RNAs and post-translational modifications—collectively producing the well-documented decline in milk yield, composition, and mammary cell functionality characteristic of thermal challenge in dairy cattle [31,32,33,34]. The summary of genetic markers associated with heat resistance in bovine mammary glands has been presented in Table 2. The candidate markers compiled in Table 2 are presented as a qualitative summary of the existing literature rather than as a quantitatively validated set, as the underlying studies differ substantially in design, reporting standards, and replication status.

### 4.1. Transcriptional Stress Responses, HSP Induction, and Disruption of Milk Protein Synthesis

At the transcriptional level, the most consistently documented response across both in vitro and in vivo models is the robust upregulation of heat shock protein (HSP) genes, which represents the cell’s primary defense against protein misfolding under thermal challenge. Transcriptomic analysis of mammary tissue from Holstein cows exposed to experimentally induced HS revealed 54 upregulated genes, prominently featuring *HSPA1A* and *HSPH1*, alongside stress-responsive genes *HSPA4L*, *SPINK4*, and *ZNF772*, while genes involved in DNA repair (*GADD45G*, *FEN1*) and mitochondrial function (*ND6*, *ND5*) were concurrently downregulated [19]. These findings are consistent with a broader pattern in which cellular energy is redirected from biosynthetic and maintenance functions toward mitigating acute stress. Complementary transcriptomic profiling of lactogenic MAC-T cells confirmed that HS triggers coordinate upregulation of HSP genes across multiple molecular weight families—including *HSPA6* and *HSPA1A* (HSP70, family), *DNAJB1*, (HSP40), *HSPH1* (HSP110), *HSPB1* (HSP20), and *HSP90AA1*—driven principally by the transcription factors *HSF1* and *HSF4*, with KEGG pathway analyses confirming enrichment of chaperone-mediated protein folding and endoplasmic reticulum (ER) protein processing pathways [20]. It is important to recognize that the MAC-T cell line, although a validated lactogenic model, represents a clonal epithelial monoculture lacking the stromal-epithelial cross-talk, immune cell interactions, vascularization, and systemic endocrine signaling that shape mammary HS responses in vivo; consequently, MAC-T-derived data tend to capture cell-autonomous chaperone induction and translational suppression more cleanly than tissue-level studies, whereas in vivo experiments additionally integrate feed-intake-driven substrate limitation, blood flow redistribution, and inflammatory signaling, often producing broader transcriptomic disturbance and more variable effect sizes. Throughout this review, we therefore distinguish between cell-autonomous mechanisms (drawn primarily from MAC-T studies) and integrated tissue-level responses (drawn from explant and in vivo work), with the latter required to confirm nutritional efficacy before application. A notable exception to the general attenuation of HSP expression during post-stress recovery is *HSP90B1*, which maintained elevated expression through both 2 h and 6 h recovery windows, implicating a specialized and sustained role in ER stress mitigation and mammary epithelial cell survival [20]. Parallel findings from in vivo studies in Girolando cows and *Bos indicus*-derived breeds further corroborate upregulation of *HSPD1* and *HSP90AA1* in mammary cells under field HS conditions [65]. In contrast, analysis of milk somatic cells from lactating Holstein–Friesian cows identified significant upregulation of *HSPA4*, *HSPA4L*, *HSPA1L*, *HSPD1*, and *HSP90* activators *AHSA1* and *AHSA2* [66]. Collectively, these observations establish HSP induction as a universal transcriptional signature of mammary HS response, conserved across breeds, cell models, and experimental paradigms.

However, the activation of cytoprotective chaperones is insufficient to prevent the downstream consequences of prolonged thermal stress, the most critical of which is the disruption of milk protein synthesis. In MAC-T cells exposed to 42 °C for 6 h, hyperthermia significantly decreased cell viability and increased apoptosis, accompanied by upregulation of pro-apoptotic genes *BAX*, Caspase-9, and Caspase-3, as well as *HSP70* and *HSP90B1*, while the anti-apoptotic protein *BCL2* was downregulated. Critically, reduced mRNA expression was documented for casein genes (*CSN1S1*, *CSN2*, *CSN3*), amino acid transporters (*SLC7A5*, *SLC38A2*, *SLC38A3*, *SLC38A9*), and mTOR pathway genes [23]. This convergence of pro-apoptotic signaling and suppressed translational machinery mechanistically links cellular stress to reduced milk protein output. Complementarily, analysis of mammary tissue from heat-stressed cows identified 213 differentially expressed genes, including downregulation of casein genes *CSN1S1*, *CSN2*, *CSN1S2*, and *CSN3*, as well as immune-related *BoLA-DRB3*, in association with increased *HSP90B1* and *HSP70* expression [67]. These transcriptional shifts indicate a fundamental reallocation of cellular resources from lactogenic function toward stress adaptation. It is important to note, however, that the relationship between transcriptional and translational regulation of milk protein synthesis is not always concordant. One in vivo study reported that despite significant HS-induced reductions in milk yield and protein output, the mRNA abundances of casein genes *CSN2* and *CSN3* and the transcription factor *STAT5B* remained unchanged in milk somatic cells under mild HS conditions [68]. This apparent discrepancy is resolved by evidence that HS impairs milk protein synthesis principally through post-transcriptional mechanisms. Specifically, HS significantly decreased phosphorylation of *STAT5* and *S6K1*—key regulators of milk protein synthesis and cellular growth—while increasing abundance of LC3-II, a marker of autophagy, without altering gene expression of key transporters or synthetic genes including *GLUT1*, *GLUT8*, *CSN2*, *CSN3*, *LALBA*, and *FASN* [22]. These findings position disrupted intracellular signaling, rather than altered transcription, as the primary driver of impaired secretory activity under HS, a distinction with important implications for therapeutic targeting.

### 4.2. Metabolic Signaling, Amino Acid Transport, Lipid Synthesis, and Nutritional Interventions

Central to the post-transcriptional impairment of milk protein synthesis is the mechanistic target of rapamycin (mTOR) signaling pathway, which integrates nutrient availability, energy status, and growth factor signals to regulate translation initiation. Under HS, protein abundance of *mTOR*, *AKT*, *EIF4EBP1*, and phosphorylated *EIF4EBP1* was reduced in MAC-T cells, with lower phosphorylated *EIF4EBP1* mechanistically diminishing translation initiation [69]. The complexity of *mTOR* regulation under HS is, however, context-dependent. A metabolomic investigation of MAC-T cells at 42 °C for 12 h identified 417 differential metabolites and found that, in contrast to the above, HS upregulated *mTOR* pathway genes (*mTOR*, *AKT*, *RHEB*, *eIF4E*, *eEF2K*) while downregulating inhibitory regulators *TSC1* and *TSC2*, alongside increased expression of the casein gene *CSN1S2*, suggesting that mammary epithelial cells possess a capacity to partially resist HS damage and sustain milk protein synthesis through enhanced intracellular amino acid absorption and *mTOR* activation [70]. This apparent contradiction in mTOR responses can be reconciled by considering three experimental variables that differ systematically across studies. First, thermal severity matters: moderate HS (≈39–40 °C, sub-lethal) appears to mobilize a transient compensatory upregulation of mTOR pathway components, consistent with cells attempting to sustain anabolic activity, whereas more severe or prolonged HS (≥42 °C, >6–12 h) shifts the balance toward translational suppression, dephosphorylation of S6K1 and 4E-BP1, and pro-apoptotic signaling. Second, cellular context modifies the response: undifferentiated MAC-T cells lack the lactogenic signaling milieu of hormonally primed or differentiated cells. They may therefore yield different mTOR readouts than those observed in mammary explants or in vivo tissue. Third, the choice of mTOR readout is decisive—mRNA abundance, total protein, and phosphorylated active forms can move in opposite directions, and studies that report only one of these captures only part of the picture. Once these variables are acknowledged, the literature is more consistent with a biphasic, intensity- and duration-dependent mTOR response than with a qualitative disagreement among studies, reinforcing the need to standardize experimental HS protocols. The pro-apoptotic regulation of *mTOR* under HS is further modulated by *CASTOR1*, a negative regulator of the pathway whose expression is increased by HS; silencing *CASTOR1* inhibited HS-induced apoptosis, promoted proliferation, and enhanced milk component synthesis, partly through facilitating *SREBP1* nuclear transport for lipid synthesis [71]. Furthermore, *HSF1* itself appears to occupy a dual regulatory role in lactogenic function: beyond its canonical function as a transcriptional activator of HSPs, *HSF1* participates in the *AKT*/*mTOR* signaling axis through direct protein interaction with *AKT*, and additionally regulates transcription of *CPSF1*, which in turn modulates *SREBP1* expression and milk fat synthesis [72]. These findings establish *HSF1* as a molecular nexus connecting the acute stress response to chronic perturbations in milk biosynthesis.

Amino acid transport represents a critical node through which HS constrains substrate availability for milk protein synthesis. The solute carrier family transporter *SLC7A5*, an essential large neutral amino acid transporter, was consistently downregulated under HS across differentiated MAC-T cells, undifferentiated MAC-T cells, and in vivo mammary tissue [20,23]. At the same time, *SLC38A2*, *SLC38A3*, and *SLC38A9* were similarly suppressed [23]. The consequences of impaired amino acid transport extend beyond substrate limitation to encompass metabolic reprogramming of intracellular amino acid pools. Metabolomics under HS identified significant perturbations in glutathione, phenylalanine, tyrosine, tryptophan, branched-chain amino acids, and several other amino acid metabolic pathways [70]. In contrast, reduced milk concentrations of methionine, phenylalanine, tyrosine, and branched-chain amino acids in heat-stressed cows corroborated these in vitro findings at the systemic level [73]. These observations collectively suggest that HS engages a coordinated suppression of amino acid uptake and metabolic utilization that synergizes with impaired *mTOR* signaling to restrict milk protein synthetic capacity.

Nutritional interventions targeting amino acid supply have shown considerable promise in reversing the transcriptional and translational impairments induced by heat stress (HS). Methionine (Met) supplementation during HS reduced phosphorylated eukaryotic elongation factor 2 abundance, upregulated *HSPA1A* (approximately 1.5-fold vs. control, *p* < 0.001), and restored HS-induced reductions in mRNA abundance of key transcriptional and translational regulators (*MAPK1*, *MTOR*, *SREBF1*, *RPS6KB1*, *JAK2*), insulin signaling genes (*AKT2*, *IRS1*), amino acid transporters (*SLC1A5*, *SLC7A1*), and the proliferation marker *MKI67* (2.5- to 3-fold increase, *p* < 0.05) [69]. In a dose–response study, Met supplementation at 20 μg/mL under hyperthermic conditions exerted cytoprotective and lactogenic effects, upregulating the anti-apoptotic gene *BCL2* (~1.8-fold, *p* < 0.05) while downregulating pro-apoptotic *BAX*, *CASP3*, and *CASP9* (40–60% reduction, *p* < 0.05) and enhancing casein gene expression (*CSN1S1*, *CSN2*) through AKT–RPS6KB1–RPS6 signaling [74]. Adjusting the lysine-to-methionine ratio to 2.5:1 elicited broader, multi-tiered responses, further upregulating casein genes and anti-apoptotic factors while engaging stress-response (*BAG3*, *DNAJB1*) and Wnt signaling pathways [75].

Arginine supplementation reversed the majority of HS-induced transcriptional changes and reduced phosphorylated EIF4EBP1 (*p* < 0.05), suggesting regulatory effects on translation initiation that are independent of mTOR signaling [69]. Combined leucine and sodium acetate supplementation similarly attenuated HS-induced cellular damage, reducing *HSP70* and *BAX* expression while enhancing β-casein synthesis [76]. At the in vivo level, rumen-protected methionine (RPM) supplementation during HS increased mammary phosphorylated mTOR and the p-EEF2/EEF2 ratio by approximately 50–60% (*p* = 0.04), translating in vitro findings to a physiologically relevant context. However, RPM did not attenuate LPS-induced inflammatory gene expression in mammary explants (*p* > 0.10), indicating that its benefits are confined to translational regulation rather than immune modulation [77]. Taurine supplementation (50–100 mM) conferred cellular protection by suppressing HSF1 nuclear translocation, enhancing antioxidant enzyme activity (SOD and GSH-PX by 1.5- to 2-fold, *p* < 0.01), and attenuating mitochondrial damage and apoptosis through downregulation of *BAX* and Caspase-3 (approximately 40–60% reduction, *p* < 0.05) [78]. Rumen-protected L-tryptophan upregulated *HSP70* and *HSP90* in peripheral blood mononuclear cells (approximately 30–65% increase, *p* < 0.05) and was associated with improved milk yield (0.3–0.5 kg/d, *p* < 0.05) and milk protein content (trend, *p* = 0.052) in lactating cows [79]. Across these interventions, Met—particularly at a Lys:Met ratio of 2.5:1—and taurine demonstrated the most consistent effect sizes across multiple endpoints, including apoptosis reduction (40–60%) and activation of synthetic pathways (1.5- to 3-fold). Arginine and tryptophan exhibited more targeted effects on translation initiation and thermotolerance, respectively. Formal quantitative synthesis across studies is precluded by heterogeneity in experimental models, intervention doses, and outcome measures; nonetheless, the consistent direction and magnitude of reported effects support the potential of targeted amino acid supplementation as a strategy to mitigate HS-induced mammary dysfunction.

Beyond apoptosis and translational regulation, HS substantially impairs milk fat synthesis through transcriptional suppression of key lipogenic genes. Transcriptome sequencing of mammary tissue identified 5705 differentially expressed genes under HS, with prominent downregulation of lipid metabolism genes *CD36*, *SLC27A6*, and *SCD* [24]. The HS-mediated downregulation of these genes, particularly *CD36* and *SLC27A6* which mediate long-chain fatty acid uptake, is mechanistically consistent with reduced milk fat yield observed in vivo [19]. At the cellular level, HS promotes apoptosis via mitochondrial dysfunction, with disruption of mitochondrial dynamics characterized by increased fission (*Drp1*, *Fis1*) and decreased fusion (*Mfn1*, *Mfn2*), alongside elevated reactive oxygen species generation; dihydromyricetin pretreatment reversed these changes, highlighting the centrality of mitochondrial integrity to mammary cell survival under thermal stress [80]. Mitochondrial protection is further conferred by *UFBP1*, a component of the ufmylation pathway, whose overexpression attenuated HS-induced mitochondrial dysfunction—evidenced by increased mitochondrial membrane potential, ATP synthesis, and NAD+/NADH ratio with reduced ROS—and restored expression of milk fat and protein-related genes in heat-stressed bovine mammary epithelial cells [81].

### 4.3. Non-Coding RNA Networks and Epigenetic Regulation of Mammary Gene Expression

The regulation of mammary gene expression under heat stress (HS) extends beyond the protein-coding transcriptome to encompass a complex landscape of non-coding RNA-mediated post-transcriptional control. For clarity, the key findings can be grouped into four mechanistic themes: (i) HS-altered miRNA expression profiles converge on a small set of pathways—IL-1, MAPK/JNK, Wnt, TGF-β, Notch, and JAK-STAT—that govern proliferation, apoptosis, and lipid metabolism; (ii) specific protective miRNAs, exemplified by miR-27a-3p and miR-223, sustain mammary epithelial proliferation and milk protein synthesis under thermal challenge; (iii) circRNA–miRNA–mRNA ceRNA networks, including circ_002033 and circRNAs targeting CD36, link non-coding regulation to milk fat synthesis and apoptotic resistance; and (iv) lncRNAs and m6A epitranscriptomic marks fine-tune the expression of HSP90B1, PRLR, and apoptotic genes. The supporting details are summarized below.

Before reviewing individual ncRNA findings, it is essential to clarify the strength of evidence underpinning each candidate. The HS-responsive ncRNAs reported in the bovine mammary gland fall into four broadly defined evidence tiers: (Tier 1) differentially expressed under HS by RNA-seq or qPCR but with no functional manipulation reported; (Tier 2) functionally validated in vitro through mimic/inhibitor, overexpression, or knockdown experiments in MAC-T or primary bovine mammary epithelial cells, with measurable downstream phenotypes (proliferation, apoptosis, casein synthesis, lipogenesis); (Tier 3) functionally validated in vivo in lactating dairy cattle through targeted loss- or gain-of-function; and (Tier 4) demonstrated, in field-level HS studies, to be quantitatively associated with milk yield, fat, or protein output. To our knowledge, no ncRNA implicated in mammary HS responses to date has reached Tier 3 or Tier 4—that is, none has been validated through in vivo knockout or knockdown in dairy cattle, nor quantitatively linked to milk yield under naturally occurring field HS conditions. The bulk of the ncRNA evidence summarized below therefore corresponds to Tier 1 (descriptive) or Tier 2 (in vitro functional), and should be interpreted accordingly. For reader clarity, the evidence tier of each ncRNA candidate is stated explicitly in-text alongside its first mention below, and the full set of candidates is consolidated by tier in the integrative summary that closes this section.

Heat stress induces significant changes in microRNA (miRNA) expression profiles, with RNA sequencing of mammary tissue identifying 124 differentially expressed miRNAs in heat-stressed Holstein cows—including bta-let-7c, bta-let-7e, bta-miR-181d, bta-miR-452, and bta-miR-31 predicted to target IL-1, and bta-miR-25 and bta-miR-382 potentially influencing the MAPK pathway through *JNK* [82], while an earlier study identified 27 differentially expressed miRNAs, with bta-miR-21-5p, bta-miR-99a-5p, bta-miR-146b, bta-miR-145, and bta-miR-133a as predominantly altered, regulating Wnt, TGF-β, MAPK, Notch, and JAK-STAT signaling pathways [82]. HS also upregulated miRNAs associated with cell growth arrest and apoptosis (miR-34a, miR-92a, miR-99, miR-184) and oxidative stress (miR-141, miR-200a), while downregulating fat synthesis-related miRNAs (miR-27ab, miR-221), providing a miRNA-level explanation for the concurrent suppression of lipogenic and anti-apoptotic gene expression [69]. These miRNAs are currently supported by Tier 1 (correlative, expression-only) evidence; with the partial exceptions of miR-27a-3p and miR-223 discussed below, no functional manipulation, in vivo loss- or gain-of-function experiments, or quantitative association with milk-yield phenotype under field HS has been reported for the majority of the listed transcripts.

Extending these observations, miR-27a-3p has been shown to exert a protective role in bovine mammary epithelial cells (BMECs) under HS by preventing oxidative stress and mitochondrial damage through regulation of the balance between mitochondrial fission and fusion processes; moreover, miR-27a-3p promotes cell proliferation via activation of the MEK/ERK pathway and upregulation of cyclin D1/E1, and also modulates milk protein synthesis-related factors including *CSN2* and *ELF5*, with pharmacological inhibition of MEK/ERK signaling by AZD6244 abolishing these proliferative and lactogenic effects, collectively demonstrating a mechanistic link between miR-27a-3p, the MEK/ERK axis, and the attenuation of HS-induced apoptosis and lactation defects in BMECs [83]. This evidence is Tier 2 (in vitro functional, BMEC-based), with mechanistic support from pharmacological MEK/ERK inhibition; however, no in vivo loss- or gain-of-function experiments in lactating cows have been performed, and the relationship of mammary miR-27a-3p abundance to milk-yield decline under natural HS in the field remains untested.

Milk-derived exosomal miRNAs have additionally been implicated in modulating mammary gland resistance to HS and enhancing milk synthesis [84], and miR-223—significantly downregulated under HS in MAC-T cells—was shown to promote cell proliferation by targeting *PRDM1*, a transcriptional repressor whose HS-induced upregulation inhibits proliferative signaling, with miR-223 mimics or *PRDM1* knockdown partially reversing heat-stress-induced proliferative inhibition [85]. This represents Tier 2 in vitro evidence in MAC-T cells; the in vivo functional role of miR-223 in lactating cattle and its quantitative association with field-level milk yield under HS have not been examined.

These miRNA networks are further embedded within circular RNA (circRNA)-mediated competing endogenous RNA (ceRNA) architectures, wherein a novel circRNA, circ_002033, significantly upregulated in heat-stressed mammary tissue, acts as a molecular sponge for miR-199a-5p to de-repress *MAP3K11* expression, with its knockdown elevating miR-199a-5p levels, reducing *MAP3K11* expression, and enhancing cellular proliferation and resilience against apoptosis and oxidative damage [86]. This is Tier 2 in vitro evidence (BMEC knockdown); in vivo functional confirmation and field-level milk-yield association remain to be demonstrated. Transcriptomic profiling further identified novel_circ_011229, bta-miR-335, and bta-miR-129 as components of ceRNA networks regulating *HSP90B1*, *PRLR*, and IGF-1 expression, with KEGG analyses implicating MAPK, apoptosis, autophagy, and prolactin signaling pathways as downstream effectors [32], while circRNA–miRNA–mRNA networks involving *circFCHSD2*, *circHNRNPLL*, *circKANSL1*, and *circMAP7* were shown to regulate *CD36* expression through miR-6516, miR-11986b, miR-345, and miR-502b, connecting circRNA biology to milk fat synthesis under HS [24]. These latter networks were inferred from transcriptomic co-expression and computational ceRNA prediction rather than from experimental knockdown or overexpression, and therefore represent Tier 1 (in silico/correlative) evidence pending direct functional validation.

Broader reviews of non-coding RNA function in ruminants further confirm that miRNAs play central roles in regulating milk fat synthesis and catabolism, and that lncRNAs and circRNAs act on milk fat-related genes through indirect interactions with miRNAs within ceRNA networks, though precise regulatory mechanisms and the extent of positive or negative self-regulatory feedback remain areas requiring further investigation [87]. At the lncRNA level, RNA sequencing of mammary tissue identified 202 differentially expressed lncRNAs under HS, with target genes enriched in apoptosis, MAPK, AMPK, mTOR, and prolactin signaling pathways; ceRNA network analyses further revealed that *HSP90B1* expression is regulated by the lncRNA *MSTRG.8646.1* through competitive sequestration of miR-152-z, while *PRLR* is modulated by *MSTRG.6147.5* and *MSTRG.8643.1* via bta-miR-335 [31], collectively delineating a multilayered post-transcriptional regulatory framework through which HS orchestrates mammary epithelial dysfunction, impaired milk synthesis, and altered cell fate decisions. These lncRNA-miRNA-mRNA axes are currently supported by computational ceRNA prediction and correlative co-expression (Tier 1); no in vitro or in vivo experimental knockdown of these lncRNAs has been reported in bovine mammary cells, and their causal contribution to milk-synthesis phenotypes therefore remains to be established.

Environmental HS during the dry period downregulated a specific lncRNA containing seven miRNA seed regions potentially governing 1159 downstream target genes, including *SOCS3*, *IGF1R*, *PPARGC1A*, *ACACA*, *VEGFA*, and *ERBB2*, alongside upregulation of immune and inflammatory genes and downregulation of ductal branching morphogenesis genes, providing molecular context for the 4.8 kg reduction in subsequent-lactation milk yield documented in heat-stressed dry cows [88]. This dry-period study represents one of the few datasets in which mammary ncRNA dysregulation has been reported alongside a quantitative subsequent-lactation milk-yield deficit at the herd level; however, the causal link between the implicated lncRNA and the milk-yield phenotype remains correlative, as no targeted ncRNA manipulation was undertaken to test causality.

At the epitranscriptomic level, N6-methyladenosine (m6A) RNA modification represents an additional regulatory mechanism through which HS modulates gene expression; conjoint analysis of m6A modification and gene-expression profiles in MAC-T cells identified multiple genes that were both differentially expressed and differentially methylated, including *HSP70*, *HSF1*, *BAX*, and *CASP3*, establishing m6A as a functional regulator of the mammary HS transcriptome [33]. The m6A evidence is likewise Tier 1/Tier 2—derived from MAC-T transcriptome-wide methylation profiling with limited downstream functional perturbation—and awaits in vivo confirmation in lactating mammary tissue and association testing against field milk-yield data.

Taken together, the ncRNA layer of the mammary HS response is at present richly characterized at the transcriptomic and computational-network level, but mechanistically anchored by only a small number of in vitro functional studies—chiefly miR-27a-3p, miR-223, and circ_002033—all of which were conducted in MAC-T cells or primary BMECs rather than in lactating animals. To our knowledge, no ncRNA has yet been targeted by in vivo knockout or knockdown in dairy cattle under HS, and no ncRNA has been quantitatively associated with field-level milk yield, fat, or protein output in cows exposed to naturally occurring thermal challenge. The candidate ncRNAs catalogued in this section should therefore be interpreted as biologically plausible regulators of mammary HS responses awaiting in vivo and field-phenotypic validation, rather than as established functional determinants of milk-production loss under heat stress. Closing this in vitro-to-in vivo gap—through ncRNA-targeted antagomir or agomir interventions in lactating cattle, sponge-RNA approaches, or quantitative association studies linking circulating or mammary-tissue ncRNA abundance to milk-yield decline under field HS—represents the most important next step for translating this regulatory layer into selection or therapeutic application.

In summary, the ncRNAs discussed in this section fall into four evidence tiers. Tier 1 (correlative; differential expression only) includes most reported miRNAs (e.g., bta-miR-21-5p, bta-miR-146b, miR-27ab, miR-221, miR-34a, miR-141), the circRNA networks involving circFCHSD2, circHNRNPLL, circKANSL1, and circMAP7, the lncRNAs MSTRG.8646.1, MSTRG.6147.5, and MSTRG.8643.1, and the m6A-modified HSP70, HSF1, BAX, and CASP3 transcripts. Tier 2 (in vitro functional validation) is limited to miR-27a-3p, miR-223, and circ_002033. Tier 3 (in vivo validation in dairy cattle) and Tier 4 (field-level association with milk yield): none reported to date. Thus, most ncRNA evidence remains correlative, with only a few functionally validated candidates, and the absence of in vivo and field-level data represents the main limitation for translational application.

### 4.4. Temporal, Endocrine, and Genomic Architecture of Thermotolerance

The timing of HS exposure is also important, as HS during the non-lactating dry period has enduring effects on subsequent lactation performance by altering mammary gland involution and redevelopment. Cooling during the early dry period upregulated genes related to apoptosis (*CASP3*), autophagy (*BECN1*, *ATG3*, *ATG5*), and heat shock response (*HSP90*, *HSF1*) compared to HS, while heat-stressed cows exhibited reduced mammary cell apoptosis and proliferation, delayed involution, and decreased connective tissue deposition, indicating that HS extends involution by suppressing the programmed cell death required for efficient gland remodeling [89]. These dry period effects propagate into early lactation through compromised *mTOR* signaling: dry period-HT cows produced significantly less milk with lower protein yield and content in the subsequent lactation, exhibiting increased inhibitory 4E-BP1 and decreased activating *Akt* and p70 *S6K1*, indicating reduced protein synthesis capacity [90]. During mammary redevelopment at day 35 of the dry period, pro-apoptotic genes (*BAX*, *AIFM1*, *IGFBP3*, *IGFBP5*) were upregulated, and *PRLR* (long form) was elevated. At the same time, *ESR2* was downregulated in heat-stressed cows, reflecting altered hormonal signaling during a critical phase of secretory tissue development [91]. Serotonin signaling genes, including the pro-apoptotic *FASLG*, were dynamically altered by HS during involution, and 5-HTP treatment upregulated the cell survival gene *FOXO3* under HS, suggesting that serotonergic modulation of epithelial cell turnover may offer a strategy to mitigate the effects of dry-period HS on subsequent lactation [92].

Endocrine regulation of mammary function under heat stress adds a systemic dimension to the cellular mechanisms described above. Prolactin (*PRL*) and its receptor *PRLR*, along with thyrotropin-releasing hormone (*TRH*), were identified as candidate genes associated with milk performance, reproduction, and heat stress response. In 1152 Chinese Holstein cows, 13 SNPs across *TRH* (8), *PRL* (3), and *PRLR* (2) were genotyped. Population allele frequencies for key SNPs in *TRH* were g.55888602A/C (A = 0.811, C = 0.189) and g.55885455A/G (A = 0.621, G = 0.379). Association analyses revealed significant associations (*p* < 0.05) for all 13 SNPs with at least one trait; a Bonferroni correction was applied to control for multiple testing. However, the study did not report effect sizes or standard errors, and no independent replication cohort was analyzed. Thus, while these SNPs are promising molecular markers, validation in additional populations with full reporting of effect sizes is required [93]. These hormonal regulatory genes intersect with the ceRNA networks described above, as lncRNA-miRNA axes regulate *PRLR* under HS [31]. Its upregulation in Girolando cows under field HS conditions, concurrent with upregulation of *INSR* and downregulation of *NR3C1*, suggests that HS recalibrates the hormonal sensitivity of the mammary gland in ways that may have both adaptive and maladaptive consequences [65].

At the population genetic level, the antagonistic genetic relationship between thermotolerance and milk production constitutes an important constraint on the breeding strategies discussed below. Although individual SNPs (for example *HSPA4* g.44660065 C>T, HSP70A1A g.-133 G>C) have been reported as associated with heat tolerance with no detected adverse effect on yield in their respective study populations, this should not be extrapolated to imply that the polygenic architectures of the two traits are independent. At the whole-population level, statistically significant negative genetic correlations between heat-tolerance phenotypes (slope of milk-yield decline above the THI threshold, rectal temperature, respiratory rate) and 305-day milk, fat, and protein yield have been documented across multiple Holstein-Friesian populations, with reported r_g estimates typically falling between approximately −0.20 and −0.45. In Australian Holsteins, national genomic-evaluation studies have estimated genetic correlations between heat-tolerance breeding values and milk-yield breeding values in this range, with steeper antagonisms detected for fat and protein yield than for milk volume. Comparable values have been reported for US Holsteins under the genomic-selection era, for European Holsteins evaluated against THI-conditional reaction-norm models, and within the Thai–Holstein population analyzed by Boonkum et al. [43], where the genetic correlation between milk yield and the fat-to-protein ratio under HS was −0.24 and the slope of yield decline was positively associated with the proportion of Holstein ancestry. The implications are twofold. First, the historical intensification of single-trait selection on milk yield in temperate environments has plausibly contributed to the elevated thermosensitivity now observed in high-merit Holstein–Friesian populations. Second, single-trait selection on thermotolerance in isolation would risk material erosion of genetic merit for production. The defensible breeding strategy is therefore a multi-trait genomic-selection index that explicitly weights thermotolerance and production traits jointly, with index weights calibrated to the projected thermal load of the target production environment. Reaction-norm and random-regression models, in which sire breeding values vary continuously along the THI gradient, provide the appropriate statistical framework and increasingly underpin national genomic evaluations for heat tolerance in Australia and the United States. Building on this population-genetic context, genome-wide association and candidate gene studies have begun to delineate the genomic architecture of thermotolerance in dairy cattle [94]. Thermal challenge activates a coordinated transcriptional program involving key regulators such as *HSF1*, *MAPK8IP1*, and *CDKN1B*, which collectively mediate chaperone-mediated protein folding, cell cycle arrest, and pro-apoptotic and oxidative stress signaling. These molecular perturbations converge to reduce milk, fat, and protein yields, with effects that are particularly pronounced in high-producing cows whose elevated metabolic demands amplify thermosensitivity, as well as in older animals with diminished cellular resilience. The well-documented negative genetic correlation between peak lactation performance and thermotolerance underscores the biological trade-off inherent to intensive dairy selection, wherein genomic regions that favor high yield also confer greater vulnerability to heat stress. Elucidating these gene regulatory pathways provide a mechanistic foundation for marker-assisted and genomic selection strategies aimed at developing thermotolerant cattle breeds capable of sustaining productive performance under the increasingly challenging thermal environments associated with global climate change [27]. GWAS in 300 lactating Holstein cows in a hot and humid semiarid environment identified SNPs within *TLR4*, *GRM8*, and *SMAD3* as significantly associated with milk yield, rectal temperature, and respiratory rate, with rectal temperature explaining 36.2% of the variance in milk yield in cows with multiple favorable genotypes [26]. Polymorphisms in the 5′ regulatory and translated regions of the heat shock protein 70 kDa protein–A1 (*HSP70A1A*) have been associated with cellular protection against heat stress and thermotolerance in dairy cows. In Chinese Holstein cattle, three SNPs were identified in 149 multiparous lactating cows via direct sequencing. A luciferase reporter assay revealed that the GG genotype at g.-133 increased promoter activity compared to the wildtype CC. Furthermore, the coding SNP g.462 G>T, resulting in an amino acid substitution (Gln154His), was predicted by DNAStar Protean software (Lasergene v17.4.0, DNAStar Inc., Madison, WI, USA) to alter protein hydrophilicity and secondary structure. Despite these observations, direct functional validation—including protein stability assays, chaperone activity tests, or cellular thermotolerance phenotypes in engineered cell lines—remains absent. Consequently, while these *HSP70A1A* polymorphisms represent promising candidate markers for thermoregulation, their mechanistic contribution to heat tolerance requires rigorous experimental confirmation prior to application in genetic selection programs [29]. In Chinese Holsteins, *EIF4E*, *HSPA4*, and *ITPR2* were identified as heat stress response determinants; five *HSPA4* SNPs, including the 3′ UTR variant g.44660065 C>T (no protein structure change), were associated with rectal temperature (*p* < 0.05; allele frequencies C = 0.72, T = 0.28). However, effect sizes, standard errors, replication status, and multiple testing correction were not reported. The variant showed no association with milk traits, suggesting utility for thermotolerance selection without yield loss, though independent validation is needed [28]. Collectively, the emerging genomic architecture of thermotolerance encompasses stress-response genes, immune regulators, and signaling-pathway components, reflecting the polygenic and pleiotropic nature of adaptation to thermal challenge in the bovine mammary gland.

### 4.5. Genotype-by-Environment Interactions and Cross-Population Transferability of Heat-Tolerance QTL

A frequently underemphasized feature of the genomic architecture of thermotolerance is that QTL and SNP effects are not invariant across production environments. Genotype-by-environment (G × E) interaction occurs when the same allele produces different phenotypic effects under different climates, management systems, or thermal-load distributions, and is well documented for heat-tolerance traits in dairy cattle. SNPs and candidate genes identified as significantly associated with milk-yield resilience or thermoregulatory phenotypes in temperate Holstein populations frequently show attenuated, absent, or oppositely signed effects when tested in tropical or subtropical *Bos taurus*, *Bos indicus*, or composite populations, and the converse is also true. For example, *HSP70A1A* promoter and coding variants identified in Chinese Holsteins (g.-133 G>C; g.462 G>T) [28] have not been reproducibly associated with thermotolerance phenotypes in field cohorts of tropically adapted *Bos indicus* or zebu-cross populations such as Sahiwal, Gir, or Girolando, where the dominant heat-adaptive loci appear to lie in different chromosomal regions and to involve coat-colour, sweat-gland, and slick-hair pathway genes rather than HSP-family members. A particularly well-documented example of an environment-dependent SNP effect is the SLICK1 variant of the prolactin receptor (*PRLR* c.1382delC; p.Leu462*) on BTA20, originally identified in tropically adapted Senepol cattle and subsequently introgressed into Holstein backgrounds: carriers of the truncated allele exhibit short, sleek coats and significantly lower rectal temperatures, sustaining milk yield under high-THI conditions, while displaying no measurable advantage—and in some studies a slight production penalty—under thermoneutral conditions, providing a clear instance of an allele whose phenotypic effect is conditional on the thermal environment [95,96]. Comparable reaction-norm QTL mapping in U.S. Holsteins has further identified *HSPA8*- and *SOD1*-region SNPs whose allele substitution effects on milk yield switch sign between low- and high-THI strata, confirming that environment-dependent SNP effects in dairy cattle are not anecdotal but reproducibly detectable when reaction-norm designs are applied. Reaction-norm and random-regression models, in which sire breeding values are allowed to vary as a continuous function of THI, provide the appropriate statistical framework for quantifying G × E for heat tolerance, and have been deployed in national genomic evaluations in Australia, the United States, and several European systems; in these analyses, the genetic correlation of breeding values estimated at low THI versus high THI typically declines well below unity (often 0.6–0.8 for milk yield, lower for component traits), indicating non-trivial reranking of sires across the thermal gradient. The breeding-programme implications are substantial. First, heat-tolerance breeding values estimated in temperate Holstein reference populations cannot be transferred wholesale to tropical or subtropical production systems, because the SNP–phenotype relationships and the underlying biology of adaptation differ. Second, multi-environment training sets that span the THI gradient relevant to the target population, and where possible multi-breed reference cohorts that include indigenous tropical breeds, are required for genomic prediction of heat tolerance to retain accuracy outside its training environment. Third, the practical deployment of any single SNP or polygenic score as a heat-tolerance marker should be preceded by validation in the specific production environment of intended use, since temperate-derived markers carry no guarantee of tropical performance.

**Table 2:** Qualitative overview of candidate genetic and molecular markers reported to be regulated in the bovine mammary gland under heat stress.

Gene/Marker	Category	Expression Status	Functional Role	Key Mechanistic Insight	Reference
*HSPA1A*, *HSPA6*, *HSPH1*	Heat Shock Protein genes	Upregulated under HS	Chaperone-mediated protein folding; ER stress mitigation	Universal HS transcriptional signature across breeds	[19,20]
*FASN*, *ACACA*, *SCD*, *CD36*, *SLC27A6*	Lipogenic/fatty acid metabolism genes	Downregulated under HS	De novo lipogenesis; long-chain FA uptake; milk fat synthesis	SCD and CD36 regulated by circRNA–miRNA networks under HS	[19,24]
*SLC7A5*, *SLC38A2*, *SLC38A3*, *SLC38A9*	Amino acid transporter genes	Downregulated under HS	Large neutral amino acid transport; substrate supply for milk protein synthesis	Consistent downregulation across MAC-T and in vivo models	[20,23]
*HSF1*, *HSF4*	Heat shock transcription factors	Upregulated and activated under HS	Transcriptional activation of HSP genes; AKT/mTOR interaction; CPSF1/SREBP1 regulation	HSF1 links acute stress to chronic milk fat/protein synthesis impairment	[20,72]
*HSP90B1*	Heat Shock Protein 90 family	Sustained upregulation (2 h & 6 h recovery)	ER stress mitigation; mammary epithelial cell survival	Regulates via ceRNA: lncRNA MSTRG.8646.1/miR-152-z axis	[20]
*CSN1S1*, *CSN2*, *CSN3*, *CSN1S2*	Casein milk protein genes	Downregulated under HS	Milk protein synthesis; casein micelle formation	Transcriptional suppression; post-transcriptional impairment via STAT5/S6K1	[23,67]
*BAX*, *CASP3*, *CASP9/BCL2*	Apoptosis regulatory genes	BAX/CASPs upregulated; BCL2 downregulated	Mitochondrial apoptotic pathway; cell viability	Methionine supplementation reverses apoptotic gene changes	[23,74]
circ_002033, circFCHSD2, circHNRNPLL	Circular RNAs	Upregulated under HS	ceRNA sponge for miR-199a-5p/miR-6516; regulates MAP3K11, CD36 expression	Knockdown of circ_002033 enhances cellular resilience	[24,86]
*TLR4*, *GRM8*, *SMAD3*	Immune/signaling pathway genes	SNP-associated with HS traits	Rectal temperature, respiratory rate, milk-yield association	Rectal temperature explains 36.2% of variance in milk yield	[26]
*HSPA4* (g.44660065 C>T in 3′ UTR)	HSPA4 gene SNP	Associated with rectal temperature; no effect on milk traits	Thermotolerance without compromising milk production	Alters mRNA secondary structure and miRNA binding	[28]
*HSP70A1A* (g.-133G>C; g.462G>T)	Heat shock protein gene variants	Promoter SNP increases activity; coding SNP alters structure	Chaperone function; thermotolerance marker	GG genotype at g.-133 → increased promoter activity; 154 Gln→His substitution	[29]
*PRLR*	Endocrine signaling receptor	Upregulated in Girolando under field HS	Mediates prolactin-driven lactogenesis; recalibrates hormonal sensitivity	Regulated by lncRNA-miRNA ceRNA axes (MSTRG.6147.5/bta-miR-335)	[31,65]
m6A-modified: *HSP70*, *HSF1*, *BAX*, *CASP3*	Epitranscriptomic (m6A) markers	Both differentially expressed and m6A-methylated under HS	N6-methyladenosine regulation of HS transcriptome	m6A is a functional regulator of mammary HS gene expression	[33]
bta-miR-21-5p, bta-miR-146b, miR-223, miR-27ab	MicroRNAs	Differentially expressed under HS	Post-transcriptional regulation of Wnt, TGF-β, MAPK, JAK-STAT; proliferation, fat synthesis	miR-223 targets PRDM1; miR-27ab regulates lipid synthesis genes	[34,69,85]
*mTOR*, *AKT*, *EIF4EBP1*, *RPS6KB1*	mTOR signaling pathway genes	Reduced protein abundance/phosphorylation under HS	Translation initiation; milk protein synthesis regulation	RPM supplementation restores p-mTOR in mammary tissue	[69,77]

Take-home patterns from Table 2: the markers compiled therein cluster into four interpretable functional groups. (i) Cytoprotective machinery (HSPA1A, HSPA6, HSPH1, HSP90B1, HSF1/HSF4) is consistently up-regulated across breeds, cell models, and tissues, and represents the most reproducible molecular signature of mammary HS. (ii) Lactogenic gene networks (*CSN1S1–CSN3*, *FASN*, *SCD*, *CD36*, *SLC27A6*, *SLC7A5*, *SLC38A2/3/9*) are correspondingly suppressed, providing the mechanistic link between thermal challenge and reduced milk yield, fat, and protein. (iii) Translational/apoptotic regulators (*mTOR*, *AKT*, *EIF4EBP1*, *RPS6KB1*, *BAX*, *CASP3/9*, *BCL2*) define the post-transcriptional bottleneck that nutritional and pharmacological interventions—most notably methionine, arginine, taurine, and rumen-protected amino acids—partially relieve. (iv) Genomic markers and post-transcriptional regulators (*HSP70A1A*, *HSPA4*, *TLR4*, *GRM8*, *SMAD3* SNPs; ncRNA—ceRNA—m6A networks) provide the inter-individual modifier layer that translates molecular biology into selectable phenotypic variation. Together, these four clusters map directly onto the THI → cellular stress → gene regulation → phenotypic outcome framework introduced in Section 1, reinforcing that mitigation strategies will be most effective when they engage more than one cluster simultaneously (e.g., genomic selection on cluster iv combined with nutritional support of cluster iii).

Despite the growing list of candidate genes and SNPs implicated in heat tolerance in dairy cattle (Table 2), it is important to emphasize that most of these markers still await rigorous validation before they can be reliably incorporated into genomic selection programs. In particular, many reported associations lack consistent reporting of effect sizes with appropriate uncertainty estimates (standard errors or confidence intervals), comprehensive characterization of allele frequencies across breeds and production environments, formal replication in independent cohorts, and demonstrated robustness to multiple testing correction at the genome-wide level. Heterogeneity in study design, sample size, genotyping platforms, phenotype definitions (e.g., THI thresholds, slope of milk decline, physiological indicators), and statistical models further complicates direct cross-study comparison. Consequently, the markers listed should be regarded as biologically plausible candidates supported by varying levels of evidence, rather than as a finalized panel suitable for immediate deployment in marker-assisted or genomic selection.

## 5. Conclusions

Heat stress represents a multidimensional threat to dairy cattle productivity, operating across physiological, transcriptomic, and genomic scales. The evidence synthesized in this review confirms that rising THI values progressively suppress milk yield and compositional quality through a coordinated molecular assault on the bovine mammary gland—encompassing HSP induction, casein and lipogenic gene suppression, mTOR-AKT-STAT5 signaling impairment, pro-apoptotic activation, and non-coding RNA-mediated post-transcriptional dysregulation. Importantly, thermal challenge during the dry period extends these consequences into the subsequent lactation by impairing mammary involution and secretory tissue redevelopment, underscoring that point-in-time yield metrics alone underestimate the true productivity cost of HS. Promising genomic markers—including SNPs in *HSP70A1A*, *HSPA4*, *TLR4*, and *PRLR*—offer tractable targets for developing thermotolerant genotypes without disproportionate sacrifice of milk yield, and strategic *Bos indicus* genomic introgression provides a complementary avenue for improving heat resilience in high-producing backgrounds. Nutritional strategies targeting methionine, arginine, and taurine supply partially restore cellular synthetic capacity and represent practical near-term mitigation tools. Nonetheless, critical limitations remain: most transcriptomic evidence derives from Holstein cattle and non-standardized in vitro models, constraining generalizability to tropical and indigenous breeds most vulnerable to future warming. Longitudinal multi-omics studies integrating genomics, epigenomics, metabolomics, and precision phenotyping under real-world HS conditions are urgently needed. Future research should prioritize multi-trait genomic selection indices that jointly optimize thermotolerance and production performance, characterize the inter-generational heritability of epigenetic HS marks, and validate candidate SNPs across diverse breed populations to accelerate the development of climate-resilient dairy cattle globally. Two further caveats deserve explicit emphasis. First, the THI itself, although operationally convenient, does not capture wind speed, solar radiation, night-time recovery, or individual variation in body condition, lactation stage, and behavior; more comprehensive indices that incorporate radiation and air-flow components, together with sensor-based individual phenotyping (rumination, body temperature, panting score), are likely to outperform the classical THI in future heat-stress research and management. Second, because the bulk of transcriptomic, ceRNA, and SNP evidence reviewed here was generated in Holstein-Friesian or Holstein-derived crossbred animals, the extent to which the same molecular signatures and SNP effects transfer to Jersey, Brown Swiss, Simmental, *Bos indicus*, and indigenous tropical breeds remains incompletely tested; multi-breed validation cohorts and harmonized HS protocols are therefore a prerequisite for translating these findings into globally applicable genomic selection tools.

## Data Availability

No new data was generated for this review article.

## Abbreviations

The following abbreviations are used in this manuscript:

*HSF1*	Heat Shock Transcription Factor 1
*HSF4*	Heat Shock Transcription Factor 4
*HSPA1A*	Heat Shock Protein Family A (Hsp70) Member 1A
*HSPH1*	Heat Shock Protein Family H (Hsp110) Member 1
*HSP70*	Heat Shock Protein 70 kDa
*HSP90B1*	Heat Shock Protein 90 Beta Family Member 1
*DNAJB1*	DnaJ Heat Shock Protein Family (Hsp40) Member B1
*CSN1S1*	Casein Alpha S1
*CSN2*	Casein Beta
*CSN3*	Casein Kappa
*CSN1S2*	Casein Alpha S2
*SLC7A5*	Solute Carrier Family 7 Member 5
*SLC38A2*	Solute Carrier Family 38 Member 2
*SLC38A3*	Solute Carrier Family 38 Member 3
*STAT5*	Signal Transducer and Activator of Transcription 5
*S6K1*	Ribosomal Protein S6 Kinase Beta-1
*mTOR*	Mechanistic Target of Rapamycin
*AKT*	AKT Serine/Threonine Kinase (Protein Kinase B)
*FASN*	Fatty Acid Synthase
*SCD*	Stearoyl-CoA Desaturase
*CD36*	CD36 Molecule (Fatty Acid Translocase)
*SLC27A6*	Solute Carrier Family 27 Member 6
*BAX*	BCL2 Associated X Protein
*CASP3*	Caspase 3
*CASP9*	Caspase 9
*BCL2*	BCL2 Apoptosis Regulator
*HSP70A1A*	Heat Shock Protein 70 Family A Member 1A (same as *HSPA1A*)
*HSPA4*	Heat Shock Protein Family A (Hsp70) Member 4
*PRLR*	Prolactin Receptor
*TLR4*	Toll-Like Receptor 4
*SMAD3*	SMAD Family Member 3
*Nrf2*	Nuclear Factor Erythroid 2-Related Factor 2 (also *NFE2L2*)
*Bos indicus*	Zebu cattle (species)
*Bos taurus*	Taurine cattle (species)
bta-miR-21-5p	*Bos taurus* microRNA 21-5p
bta-miR-146b	*Bos taurus* microRNA 146b
miR-223	MicroRNA 223
*circ_002033*	Circular RNA 002033
*circFCHSD2*	Circular RNA derived from FCHSD2 gene
*MSTRG.8646.1*	MSTRG identifier for a long non-coding RNA transcript
m6A	N6-Methyladenosine
ceRNA	Competing Endogenous RNA
GWAS	Genome-Wide Association Study
QTL	Quantitative Trait Locus
SNP	Single-Nucleotide Polymorphism
*ACACA*	Acetyl-CoA Carboxylase Alpha
*AHSA1*	Activator of HSP90 ATPase Activity 1
*AHSA2*	Activator of HSP90 ATPase Activity 2
*AIFM1*	Apoptosis Inducing Factor Mitochondria Associated 1
*AKT2*	AKT Serine/Threonine Kinase 2
*ATG3*	Autophagy Related 3
*ATG5*	Autophagy Related 5
*BAG3*	BCL2 Associated Athanogene 3
*BECN1*	Beclin 1
*BoLA-DRB3*	Bovine Leukocyte Antigen DR Beta 3
*CASTOR1*	Cytosolic Arginine Sensor For mTORC1 Subunit 1
*CDKN1B*	Cyclin Dependent Kinase Inhibitor 1B
*CPSF1*	Cleavage And Polyadenylation Specific Factor 1
*Drp1*	Dynamin-Related Protein 1 (also *DNM1L*)
*EEF2*	Eukaryotic Elongation Factor 2
*eEF2K*	Eukaryotic Elongation Factor 2 Kinase
*EIF4E*	Eukaryotic Translation Initiation Factor 4E
*EIF4EBP1*	Eukaryotic Translation Initiation Factor 4E Binding Protein 1
*ELF5*	E74 Like ETS Transcription Factor 5
*ERBB2*	Erb-B2 Receptor Tyrosine Kinase 2
*ESR2*	Estrogen Receptor 2
*FASLG*	Fas Ligand
*FEN1*	Flap Structure-Specific Endonuclease 1
*Fis1*	Fission, Mitochondrial 1
*FOXO3*	Forkhead Box O3
*GADD45G*	Growth Arrest And DNA Damage Inducible Gamma
*GLUT1*	Glucose Transporter Type 1 (also *SLC2A1*)
*GLUT8*	Glucose Transporter Type 8 (also *SLC2A8*)
*GRM8*	Glutamate Metabotropic Receptor 8
*HSP90*	Heat Shock Protein 90 kDa
*HSP90AA1*	Heat Shock Protein 90 Alpha Family Class A Member 1
*HSPA1L*	Heat Shock Protein Family A (Hsp70) Member 1 Like
*HSPA4L*	Heat Shock Protein Family A (Hsp70) Member 4 Like
*HSPA6*	Heat Shock Protein Family A (Hsp70) Member 6
*HSPB1*	Heat Shock Protein Family B (Small) Member 1
*HSPD1*	Heat Shock Protein Family D (Hsp60) Member 1
IGF-1	Insulin Like Growth Factor 1
*IGF1R*	Insulin Like Growth Factor 1 Receptor
*IGFBP3*	Insulin Like Growth Factor Binding Protein 3
*IGFBP5*	Insulin Like Growth Factor Binding Protein 5
IL-1	Interleukin 1
*INSR*	Insulin Receptor
*IRS1*	Insulin Receptor Substrate 1
*ITPR2*	Inositol 1,4,5-Trisphosphate Receptor Type 2
*JAK2*	Janus Kinase 2
*JNK*	C-Jun N-Terminal Kinase
*LALBA*	Lactalbumin Alpha
LC3-II	Microtubule-Associated Protein 1 Light Chain 3 Beta-II
*MAP3K11*	Mitogen-Activated Protein Kinase Kinase Kinase 11
*MAPK1*	Mitogen-Activated Protein Kinase 1
*MAPK8IP1*	Mitogen-Activated Protein Kinase 8 Interacting Protein 1
*Mfn1*	Mitofusin 1
*Mfn2*	Mitofusin 2
*MKI67*	Marker Of Proliferation Ki-67
*ND5*	NADH Dehydrogenase Subunit 5
*ND6*	NADH Dehydrogenase Subunit 6
*NR3C1*	Nuclear Receptor Subfamily 3 Group C Member 1
*PPARGC1A*	Peroxisome Proliferator-Activated Receptor Gamma Coactivator 1 Alpha
*PRDM1*	PR/SET Domain 1
*PRL*	Prolactin
*RHEB*	Ras Homolog Enriched In Brain
*RPS6*	Ribosomal Protein S6
*RPS6KB1*	Ribosomal Protein S6 Kinase B1
*S6K1*	Ribosomal Protein S6 Kinase Beta-1 (same as *RPS6KB1*)
*SLC1A5*	Solute Carrier Family 1 Member 5
*SLC38A9*	Solute Carrier Family 38 Member 9
*SLC7A1*	Solute Carrier Family 7 Member 1
*SOCS3*	Suppressor Of Cytokine Signaling 3
*SPINK4*	Serine Peptidase Inhibitor Kazal Type 4
*SREBF1*	Sterol Regulatory Element Binding Transcription Factor 1
*SREBP1*	Sterol Regulatory Element Binding Protein 1 (same as *SREBF1*)
*STAT5B*	Signal Transducer and Activator of Transcription 5B
*TRH*	Thyrotropin-Releasing Hormone
*TSC1*	TSC Complex Subunit 1
*TSC2*	TSC Complex Subunit 2
*UFBP1*	Ufmylation Binding Protein 1
*VEGFA*	Vascular Endothelial Growth Factor A
*ZNF772*	Zinc Finger Protein 772
*4E-BP1*	Eukaryotic Translation Initiation Factor 4E Binding Protein 1 (same as *EIF4EBP1*)
bta-let-7c	*Bos taurus* let-7c microRNA
bta-let-7e	*Bos taurus* let-7e microRNA
bta-miR-25	*Bos taurus* microRNA 25
bta-miR-31	*Bos taurus* microRNA 31
bta-miR-99a-5p	*Bos taurus* microRNA 99a-5p
bta-miR-129	*Bos taurus* microRNA 129
bta-miR-133a	*Bos taurus* microRNA 133a
bta-miR-145	*Bos taurus* microRNA 145
bta-miR-181d	*Bos taurus* microRNA 181d
bta-miR-335	*Bos taurus* microRNA 335
bta-miR-382	*Bos taurus* microRNA 382
bta-miR-452	*Bos taurus* microRNA 452
*circHNRNPLL*	Circular RNA derived from HNRNPLL gene
*circKANSL1*	Circular RNA derived from KANSL1 gene
*circMAP7*	Circular RNA derived from MAP7 gene
miR-27a-3p	MicroRNA 27a-3p
miR-27ab	MicroRNA 27ab
miR-34a	MicroRNA 34a
miR-92a	MicroRNA 92a
miR-99	MicroRNA 99
miR-141	MicroRNA 141
miR-184	MicroRNA 184
miR-200a	MicroRNA 200a
miR-221	MicroRNA 221
miR-382	MicroRNA 382
miR-502b	MicroRNA 502b
miR-6516	MicroRNA 6516
miR-11986b	MicroRNA 11986b
*MSTRG.8643.1*	MSTRG identifier for a long non-coding RNA transcript
novel_circ_011229	Novel Circular RNA 011229
